# A Longitudinal Study of Parental Solicitation, Rule-Setting, and Psychological Control as Predictors of Adolescent Disclosure across More Individualistic and More Collectivistic Countries

**DOI:** 10.1007/s10964-025-02311-8

**Published:** 2026-01-07

**Authors:** Şule Selçuk, Christy M. Buchanan, Ann T. Skinner, Jennifer E. Lansford, Dario Bacchini, Marc H. Bornstein, Lei Chang, Kirby Deater-Deckard, Laura Di Giunta, Kenneth A. Dodge, Sevtap Gurdal, Qin Liu, Qian Long, Paul Oburu, Concetta Pastorelli, Emma Sorbring, Sombat Tapanya, Laurence Steinberg, Liliana Maria Uribe Tirado, Saengduean Yotanyamaneewong, Liane Peña Alampay, Suha M. Al-Hassan

**Affiliations:** 1 Hacettepe University, Ankara, Turkey; 2 Wake Forest University, Winston-Salem, NC USA; 3 Duke University, Durham, NC USA; 4 University of Naples Federico II, Naples, Italy; 5 Eunice Kennedy Shriver National Institute of Child Health and Human Development, Bethesda, MD USA; 6 UNICEF, New York, NY USA; 7 Institute for Fiscal Studies, London, UK; 8 University of Macau, Macau, China; 9 University of Massachusetts Amherst, Amherst, MA USA; 10 Sapienza University of Rome, Rome, Italy; 11 University West, Trollhättan, Sweden; 12 Chongqing Medical University, Chongqing, China; 13 Duke Kunshan University, Suzhou, China; 14 Maseno University, Maseno, Kenya; 15 Chiang Mai University, Chiang Mai, Thailand; 16 Temple University, Philadelphia, PA USA; 17 King Abdulaziz University, Jeddah, Saudi Arabia; 18 Universidad de San Buenaventura, Bogotá, Colombia; 19 Ateneo de Manila University, Quezon City, Philippines; 20 Abu Dhabi Early Childhood Authority, Abu Dhabi, UAE

**Keywords:** Solicitation, Rule-setting, Psychological control, Disclosure

## Abstract

Although autonomy-relevant parenting practices (solicitation, rule-setting, and psychological control) have been linked to adolescent disclosure, little is known about how these practices operate across cultural contexts. Existing studies often examined these practices in isolation or relied on cross-sectional designs, limiting understanding of their unique relations over time. This study examined these associations longitudinally across eight countries differing in average individualism and collectivism, focusing on the mediating role of adolescents’ perceptions of parental warmth, neglect, and overcontrol. Participants were 1,215 adolescents (50.3% girls) assessed at ages 13, 15, and 16. Perceived psychological control predicted greater perceived neglect and overcontrol, and perceived overcontrol, in turn, significantly predicted lower disclosure; this indirect effect was significant. Neither solicitation nor rule-setting predicted disclosure over time. However, when focusing only on voluntary disclosure (excluding secrecy items), solicitation predicted greater disclosure. Findings highlight the differential impact of parenting practices on disclosure over time, with psychological control as a risk factor and solicitation potentially facilitating disclosure depending on its measurement.

## Introduction

As children move into adolescence, they spend less time under the direct supervision of their parents and other adults, meaning that parents are less likely to observe their adolescent children’s behavior directly (Smetana et al., 2015). Consequently, adolescents’ disclosure to parents about their activities and whereabouts becomes a key predictor of parental knowledge of these activities (Buchanan & Selçuk, 2024) as well as adolescent well-being (e.g., Elsharnouby & Dost-Gözkan, 2020; Kerr et al., 2010; Kapetanovic & Skoog, 2021). Identifying parenting practices that predict adolescent disclosure over time is therefore important. Although autonomy-relevant parenting practices such as solicitation, rule-setting, and psychological control have been linked to disclosure (e.g., Kerr et al., 2010; Urry et al., 2011), existing research often examines these practices in isolation (i.e., failing to include all of these autonomy-related parenting practices in the same study; e.g., Keijsers et al., 2010) or relies on cross-sectional designs (e.g, Baudat et al., 2020), limiting understanding of how they uniquely shape disclosure over time. Examining these different but interrelated parenting practices together, after controlling for stability over time and concurrent associations in a longitudinal design, would provide a better understanding of the unique association of each parenting practice with changes in adolescent disclosure over time. Moreover, although these practices have been studied extensively in single-country samples (e.g., Garthe et al., 2018; Song & Smetana, 2024), comparatively little is known about how they operate across different cultural contexts. This study examines the longitudinal associations of these three parenting practices with adolescent disclosure over three years and tests whether adolescents’ perceptions of parental warmth, neglect, or overcontrol mediate these associations across more individualistic and more collectivistic countries. Because the developmental transition into early and middle adolescence (i.e., 13 to 16 years) is one in which adolescents’ disclosure tends to decrease in both more individualistic and more collectivistic countries (Cheung et al., 2013; Keijsers et al., 2009), this developmental period was the focus of the study.

### Solicitation, Rule-Setting, and Psychological Control as Predictors of Adolescent Disclosure

Self-Determination Theory (Deci & Ryan, 2000) provides a theoretical framework for understanding why some parenting practices foster disclosure while others hinder it. This theory posits that the degree to which parental techniques promote adolescents’ volitional function will determine how effective they are. Therefore, according to Self-Determination Theory, disclosure is likely to be facilitated when parents are engaged but not overly controlling. Building on this perspective, the present study focuses on three autonomy-relevant parenting practices—solicitation, rule-setting, and psychological control—as predictors of adolescent disclosure. Solicitation refers to asking adolescents questions about their everyday life and behavior (Laird et al., 2010; Stattin & Kerr, 2000). Rule-setting is defined as setting rules that require adolescents to inform their parents about where and with whom they go and what they do (Laird et al., 2010; Stattin & Kerr, 2000). Psychological control refers to manipulative and intrusive behaviors such as love withdrawal and guilt induction aimed at making children think and behave in the ways parents desire (Barber, 1996). From a Self-Determination Theory perspective, these three autonomy-relevant parenting practices might differ in the extent to which they support or constrain autonomy, providing a theoretically grounded rationale for examining their unique and joint contributions to adolescent disclosure.

Parents of adolescents engage to varying extents in solicitation, rule-setting, and psychological control. Consistent with Self-Determination Theory (Deci & Ryan, 2000), parental solicitation that respects adolescents’ autonomy may facilitate disclosure by signaling interest and involvement without undermining their sense of choice and independence. Although inherently more directive than solicitation, rule-setting can still support autonomy when rules are developmentally appropriate, clearly communicated, and framed as supportive guidance rather than coercion. In this way, rule-setting can provide structure and boundaries that help adolescents understand expectations while maintaining a sense of predictability and fairness, which can support their emerging autonomy (see Soenens et al., 2019). Theoretically, these two parenting practices constitute two main components of parental monitoring of adolescents’ everyday life, and empirically they are positively related to one another (Stattin & Kerr, 2000). In contrast, psychological control is deemed a more invasive parenting practice compared to solicitation and rule-setting, and much research shows that it interferes with healthy autonomy development (Laird et al., 2013; Van Petegem et al., 2015).

The association of psychological control with solicitation and rule-setting differs across studies (e.g., Gaertner et al., 2010; Li et al., 2015; Selçuk et al., 2022). The mixed results suggest the possibility that associations between them could differ across families (Rodríguez-Meirinhos et al., 2020). Nevertheless, it seems important to examine the three types of autonomy-relevant parental practices in the same model controlling for one another to reveal a clearer understanding of what kind of parental behaviors might be associated with the trajectory of adolescent disclosure over the early to middle adolescent years.

Several studies have addressed the role of psychological control in adolescent disclosure, generally finding that higher levels of psychological control are associated with less disclosure (e.g., Soenens et al., 2006; Song & Smetana, 2024; but see Kearney & Bussey, 2015). In contrast, research on parental solicitation has more consistently shown positive associations with disclosure, though the findings are not uniform. Cross-sectional studies, for example, have found positive links among Swiss (Baudat et al., 2020), U. S. (Fernandez et al., 2018), and Chinese adolescents (Hawk, 2017), whereas Hawk et al. (2016) reported no such associations among Swedish parents. Longitudinal findings are similarly mixed: solicitation has been shown to predict increases in disclosure among Canadian adolescents (Hamza & Willoughby, 2011) and Dutch adolescents (only when mothers reported on both constructs, Keijsers et al., 2010). However, most longitudinal studies have not found a significant link, for example, among American adolescents (Garthe et al., 2018), Dutch adolescents when adolescents or fathers provided the reports (Keijsers et al., 2010), as well as Swedish (Kerr et al., 2010) and Northern European adolescents (Tilton-Weaver, 2014). More fine-grained approaches, such as diary methods and within-person analyses, suggest that solicitation can elicit disclosure in daily interactions, particularly with mothers (Kapetanovic et al., 2019; Solís et al., 2015). Importantly, whether solicitation encourages disclosure appears to depend on adolescents’ interpretations: solicitation is more effective when not perceived as intrusive (Baudat et al., 2020) or when parents are seen as having legitimate authority (Keijsers & Laird, 2014). These patterns highlight that the link between solicitation and disclosure might vary not only across individuals but also across cultural contexts, where norms regarding parental authority and adolescent autonomy differ – a point elaborated in the following sections.

Although rule-setting is an aspect of authoritative parenting (Baumrind, 2005), and generally recommended as effective in promoting positive behavior (Pinquart, 2017; Willoughby & Hamza, 2011), which might imply more disclosure, empirical data generally show rule-setting to be negatively related or unrelated to disclosure. A study conducted in Chile, the Philippines, and the U.S showed that rule-setting (having clear rules and expectations) was associated with less, not more, disclosure in the latter two countries and unrelated to disclosure in Chile (Darling et al., 2009). Partly in line with these findings, cross-sectional studies with Swedish adolescents (Hawk et al., 2016) and Chinese adolescents (Hawk, 2017) found that, after controlling for solicitation and snooping, rule-setting was not significantly associated with disclosure. Similarly, rule-setting also did not predict increases in disclosure in longitudinal studies with Swedish adolescents (Kerr et al., 2010), Northern European adolescents (Tilton-Weaver, 2014), and Canadian adolescents (Hamza & Willoughby, 2011).

In sum, consistent with Self-Determination Theory, the empirical data reviewed suggest that the most invasive form of control—psychological control—is reliably linked to less adolescent disclosure, whereas an alternative autonomy-relevant practice that might convey engagement or interest without excessive control – solicitation – has more potential to elicit disclosure. However, rule-setting, which is generally seen as an effective parenting practice if rules are developmentally appropriate (Fletcher et al., 2004), seems either negative or neutral with respect to encouraging disclosure. Importantly, some mixed results across studies in different countries suggest that the relations of solicitation and rule-setting with adolescent disclosure might differ in different cultural contexts, perhaps as a result of adolescents’ differential interpretations of these parental behaviors and consequently their differential influence on adolescent-parent child relationship. Therefore, the current study examined whether any unique relations between the three parenting practices and adolescent disclosure could be explained by adolescents’ perceptions of parental warmth, neglect, and overcontrol.

### The Role of Adolescents’ Perceptions of Parental Warmth, Neglect, and Overcontrol in Disclosure

Adolescents actively interpret their parents’ behaviors (Soenens & Vansteenkiste, 2020) and these interpretations can shape the quality of parent-child relationship (Hawk et al., 2009). Such interpretations of parenting practices may vary considerably across individuals and contexts (Camras et al., 2017; Kapetanovic & Skoog, 2021). Indeed, in some studies, solicitation and/or rule-setting are positively correlated with parental warmth, feeling connected to parents, and perceived importance to parents (Kapetanovic & Skoog, 2021; Selçuk et al., 2022), whereas in others, the same parenting practices predict perceptions of being intruded on and overly controlled (e.g., Hawk et al., 2016; Kapetanovic et al., 2020). Similarly, although psychological control is consistently associated with diminished feelings of connectedness with parents, reduced mother-child relationship quality, and augmented perceptions of privacy invasion, intrusiveness, and overcontrol (Kakihara et al., 2010; Laird et al., 2013; Selçuk et al., 2022; Xu et al., 2021), certain aspects of psychological control (e.g., guilt induction) are interpreted more benignly in certain countries or cultural groups (Cheah et al., 2019; Mason et al., 2004). Indeed, a longitudinal study with U.S. adolescents revealed that both mothers’ and fathers’ psychological control were linked to reduced adolescent disclosure about personal matters such as free time activities via its association with lower levels of perceived parental support (Song & Smetana, 2024).

Taken together, these findings highlight that adolescents’ perceptions of warmth, neglect, and overcontrol are not simply additional predictors of disclosure but might serve as mediating processes. Although solicitation, rule-setting, and psychological control capture adolescents’ perceptions of specific parenting behaviors, warmth, neglect, and overcontrol reflect broader interpretations of the parent–child relationship. This distinction is important because all constructs in the current study are assessed through adolescent reports; thus, the effects of autonomy-relevant practices on disclosure are likely explained by the way they shape these relational perceptions. By treating perceived warmth, neglect, and overcontrol as mediators, the present study underscores their unique contribution in clarifying why the same parenting practices might encourage disclosure in some contexts but discourage it in others, particularly across different cultural settings.

### The Role of Cultural Orientation in Perceptions of Parenting Practices

One dimension on which countries vary, and that might matter to the links between perceived parenting practices and adolescents’ perceptions of warmth, neglect, and overcontrol, is the cultural leaning toward individualism and collectivism (Hofstede et al., 2010). More individualistic cultures are, on average, more likely to emphasize personal autonomy, self-reliance, and self-actualization, and more collectivistic cultures are more likely to emphasize interdependence, conformity, family loyalty, and respect for elders including parents (Hofstede et al., 2010; Oyserman et al., 2002; Triandis, 2018). Although all countries include both individualistic and collectivistic elements and there is substantial variation in every country (see Fiske, 2002; Triandis, 2018), there are well-established average differences between countries on this dimension. Furthermore, the individualism-collectivism dimension is potentially helpful in understanding variation across countries concerning the relations between parenting practices and adolescent adjustment (Chyung et al., 2022; Pinquart & Kauser, 2018).

Among the cultural beliefs and values that might be important to interpretation of autonomy-related practices are those pertaining to the legitimacy of and respect for parental authority, and the obligation to obey parents, which are expected, on average, to be greater among adolescents in more collectivistic countries than among adolescents in more individualistic countries (Alampay, 2014; Takash & Al-Hassan, 2014). Relatedly, expectations regarding the timing of autonomy might differ, with more parental control and later adolescent autonomy being more normative in more collectivistic cultures (Darling et al., 2005; Helwig et al., 2014; Rudy & Halgunseth, 2005). For these reasons, associations of the three focal parenting practices with adolescents’ perceptions of parental warmth, neglect, and overcontrol, and consequently with adolescent disclosure might differ across the two country groups such that practices that are more autonomy-limiting are perceived more negatively ~ and more likely to elicit reactance than cooperation ~ in more individualistic countries than in more collectivistic countries.

A relevant cross-sectional vignette study conducted in Turkey, which has a more collectivistic orientation, indicated that parents’ active monitoring efforts (solicitation and rule-setting combined) were not perceived negatively (e.g., as an intrusion to their life) by adolescents (Selçuk et al., 2022). On the contrary, Turkish adolescents viewed these parenting practices as highly legitimate. In keeping with this, another cross-sectional study examining solicitation and rule-setting (together with a more intrusive type of monitoring, snooping) showed that perceived rule-setting did not relate to perceptions of privacy invasion among Chinese adolescents even though there was a modest association between more perceived solicitation and more perceptions of privacy invasion (Hawk, 2017). Research conducted in countries that rank high in individualism (e.g., the Netherlands and Sweden; Hofstede et al., 2010), in contrast, has revealed positive associations between solicitation and/or rule-setting on the one hand and feelings of privacy invasion and being overcontrolled on the other (e.g., Hawk et al., 2016; Kapetanovic et al., 2020).

Regarding associations of perceived solicitation and/or rule-setting with perceptions of parental warmth, research using one-time correlational designs or reporting only bivariate associations revealed positive associations in both more individualistic and more collectivistic countries (Fernandez et al., 2021; Keijsers et al., 2009; Selçuk et al., 2022). However, little is known about their associations over time, especially in more collectivistic countries. A longitudinal (cross-lagged) study in the U.S. indicated that more parental solicitation predicted increase in perceived maternal warmth (Garthe et al., 2018). In contrast, a longitudinal study among Swedish adolescents showed that rule-setting was not linked with perceived parental warmth in the following year after controlling for other parenting practices including some aspects of psychological control (Kakihara et al., 2010). Therefore, there is some evidence that suggests a positive longitudinal association between solicitation and parental warmth whereas rule-setting’s association with parental warmth over time is questionable. In the current study, by including several countries that differ in terms of individualistic and collectivistic dimensions and by examining the three parenting practices together with a longitudinal design, the study aims to improve understanding regarding possible differential longitudinal associations of parental solicitation and rule-setting with adolescents’ perceptions of parental warmth, neglect, and overcontrol, and their potential roles as mediators of any association with adolescent disclosure.

Compared to solicitation and rule-setting practices, less of a difference is expected across countries in the associations between perceptions of psychological control and perceptions of parental warmth, neglect, and overcontrol (and eventually with disclosure). This is because, consistent with Self Determination Theory (Deci & Ryan, 2000), existing research in both more individualistic and more collectivistic countries indicates that psychological control frustrates basic and universal needs for autonomy, relatedness, and competence (Costa et al., 2016; Selçuk & Şener, 2018; see also Soenens & Vansteenkiste, 2010). Therefore, adolescents who perceive their parents to be more psychologically controlling are expected to be more likely to perceive those parents as neglectful and overly controlling – and to be less likely to perceive them as warm – regardless of the degree of individualism and collectivism. As a result, higher psychological control is expected to predict less disclosure across the two country groups. Nonetheless, the strength of the association might differ across the two cultural groups, as past studies suggest that interpretations of and reactions to psychological control are even more negative among adolescents from more individualistic than more collectivistic backgrounds (Chao & Aque, 2009; Chen et al., 2016).

## Current Study

Prior research has examined the links between autonomy-relevant parenting practices—solicitation, rule-setting, and psychological control—and adolescent disclosure, but has rarely considered these practices simultaneously, relied largely on cross-sectional single-country designs, and thus provides limited insight into their unique longitudinal effects and potential differences across cultural contexts. The current study used longitudinal data from eight countries to examine how perceived solicitation, rule-setting, and psychological control relate to adolescent disclosure over time via adolescents’ perceptions of parental warmth, neglect, and overcontrol across more individualistic (Italy, Sweden, USA) and collectivistic countries (Colombia, Jordan, Kenya, Philippines, Thailand). Based on theory and the literature reviewed, it was hypothesized that in more individualistic countries, perceived solicitation and rule-setting would be associated positively with perceptions of overcontrol, and in turn, perceptions of overcontrol would be associated negatively with adolescent disclosure (Hypothesis 1). For more individualistic countries, no clear expectations were proposed regarding the longitudinal associations of perceived solicitation and rule-setting with perception of parental warmth as previous literature does not provide a solid prediction. It was expected that in more collectivistic countries, perceived solicitation and rule-setting would be associated positively with perceptions of warmth and negatively with perceptions of overcontrol and neglect (or have a nonsignificant association with overcontrol); in turn, perceptions of warmth would be associated positively and perceptions of overcontrol and neglect negatively with adolescent disclosure (Hypothesis 2). Finally, it was hypothesized that in both country groups, perceived psychological control would be associated negatively with adolescents’ perceptions of warmth, and positively with adolescents’ perceptions of neglect and overcontrol, and in turn, perceptions of warmth would be associated positively and perceptions of overcontrol and neglect negatively with adolescent disclosure. However, it was anticipated that these associations might be stronger in more individualistic than more collectivistic countries. (Hypothesis 3).

## Method

### Participants and Procedure

The sample consisted of 1215 adolescents (50.3% girls) drawn from the Parenting Across Cultures (PAC) project, a cross-national longitudinal project. Data from three waves including the study variables were used. Although participants were originally recruited at age 8 as part of the larger project, the current study focuses on data starting at age 13, which serves as the first time point for the present study. Adolescents were an average of 13.33 years old (*SD* = 0.79) at the initial assessment and the time interval between pairs of consecutive assessments was about two years and one year, respectively. Participants were recruited from Chiang Mai, Thailand (*n* = 120, 49.2% female), Durham, NC, USA (African Americans, *n* = 102, 52.4% female, European Americans, *n* = 110, 41.3% female, and Latinx, *n* = 99, 52.5% female), Italy (Naples, *n* = 102, 52.0% female and Rome, *n* = 111, 46.8% female), Kisumu, Kenya (*n* = 100, 60% female), Manila, Philippines (*n* = 120, 49.2% female), Medellín, Colombia (*n* = 108, 55.6% female), Trollhättan/Vänersborg, Sweden (*n* = 129, 48.8% female), and Zarqa, Jordan (*n* = 114, 47.4% female). They were recruited through socioeconomically diverse schools in each country.

University institutional review board approvals in each country were obtained prior to data collection. Adolescent participants provided assent and their parents gave written informed consent prior to each wave of data collection. The scales were translated and back-translated using a rigorous procedure (Maxwell, 1996), and adolescents completed the items in their own language. Measures were administered by research assistants either in person at participants’ homes or other locations of their choosing, over the telephone, or online, depending on the respondents’ preferences.

Countries were categorized as more collectivistic or more individualistic based on an individualism index reported by Hofstede et al. (2010; Hofstede Insights, 2021). The index ranges from 0 to 100, with lower values indicating stronger collectivistic tendencies and higher values reflecting stronger individualistic tendencies. Although individualism and collectivism are not mutually exclusive constructs, dichotomizing countries based on the midpoint of the index (50) allowed for a parsimonious grouping that highlights broad cultural contrasts relevant to the research questions. Accordingly, countries with index scores above 50 [Italy (76), Sweden (71), USA (91)] were classified as more individualistic, whereas countries with scores below 50 [Colombia (13), Jordan (30), Kenya (25), Philippines (32), Thailand (20)] were classified as more collectivistic. Such classification simplifies within-country cultural heterogeneity and should be interpreted as a heuristic for examining cross-cultural patterns rather than as a definitive categorization of countries.

A total of 653 adolescents (53.7%) were from more individualistic countries and 562 adolescents were from more collectivistic countries. Mothers (13.19 years, *SD* = 4.23) and fathers (13.14 years, *SD* = 4.39) from more individualistic countries had significantly more education on average than did mothers (12.14 years, *SD* = 4.37) and fathers (12.42 years, *SD* = 4.31), respectively, from more collectivistic countries, *t*(1161) = 4.20, *p* < 0.001, *d* = 0.24 for mother education and *t*(1040) = 2.66, *p* < 0.01, *d* = 0.17 for father education.

### Measures

Cronbach’s alpha and McDonald’s omega coefficients for each scale are presented in Tables S1a and S1b in the supplementary material. Multi-group confirmatory factor analysis results conducted to test measurement invariance of each scale are displayed in Table S2 in the supplementary material. Results revealed at least metric invariance for all scales.

#### Solicitation and rule-setting

At ages 13, 15, and 16, adolescents reported on their parents’ solicitation and rule-setting with five and six items, respectively, adapted from the Youth Knowledge, Disclosure, Control, and Solicitation Scale (Stattin & Kerr, 2000). A sample solicitation item is “How often do your parents ask you about what happened during your free time?” and a sample rule-setting item is “Must you have your parents’ permission before you go out during the weeknights?” Items were rated on a 4-point Likert scale ranging from 0 (*never*) to 3 (*always*). Items were averaged, and higher scores indicate greater solicitation and greater rule-setting.

#### Psychological control

At ages 13, 15, and 16, adolescents reported on their parents’ psychological control with three items adapted from Barber et al. (1994; Barber, 1996). A sample item is “My parents act cold and unfriendly if I do something they don’t like.” Items were rated on a 4-point Likert scale ranging from 1 (*strongly disagree*) to 4 (*strongly agree*). Items were averaged, and higher scores reflect greater psychological control (see Table S3 in the supplementary material for the item-total correlations).

#### Adolescents’ perceptions of parental warmth, neglect, and overcontrol

At ages 13 and 15, adolescents reported on their perceptions of parental warmth (eight items), neglect (six items), and overcontrol (five items) with the child version of the Parental Acceptance-Rejection/Control Questionnaire-Short Form (PARQ/Control-SF; Rohner, 2005). Sample items for each of the three scales are “My mother/father says nice things about me,” “… pays no attention to me,” and “… wants to control whatever I do,” respectively. Rothenberg et al. (2020) argued that this measure of control may actually assess parental *overcontrol* based on the content of the items (e.g., “control whatever I do”) and their finding that higher ratings on this measure, at least in some countries (e.g., Jordan, Sweden), predicted negative child outcomes. Thus, this measure might assess perceptions of overcontrol, rather than age-appropriate control. Items were rated on a 4-point Likert scale ranging from 1 (*almost never*) to 4 (*every day*), and higher scores reflect higher perceived warmth, neglect, and overcontrol. For the perception of overcontrol, two items (i.e., “… sees to it that I know exactly what I may or may not do” and “… lets me do anything I want to do (reversed)”) were excluded that did not clearly fit with other overcontrol items. Specifically, these two items had lower inter-item correlations with other overcontrol items (see Table S4 in the supplementary material for the correlations), either cross-loaded (for adolescent report for mothers) or loaded on a different factor (see Table S5 in the supplementary material for the factor analysis). Adolescents’ ratings of mothers and fathers were averaged to create overall perceptions of adolescents about their parents (see Table S6 in the supplementary material for item–total correlations of the perception of overcontrol items).

#### Adolescent disclosure

At ages 13, 15, and 16, adolescents rated their level of disclosure to their parents using five items adapted from the Youth Knowledge, Disclosure, Control, and Solicitation Scale (Stattin & Kerr, 2000). A sample item is “Do you spontaneously tell your parents about your friends (Which friends you hang out with and how they think and feel about various things)?” Items were rated on a 4-point Likert scale ranging from 0 (*never*) to 3 (*always*). Items were averaged, and higher scores reflect greater disclosure.

#### Demographic information

Parents provided demographic information including the child’s birth date and gender, and parent education at the initial assessment of the project.

### Missing Data and Attrition Analysis

Gender had no missing values, and the missing value percentage in other study variables ranged from 4% to 23.9%, with the highest rates observed in study variables evaluated at age 16 (23.9% for psychological control, 23.5% for solicitation, 23.6% for rule-setting, 23.8% for disclosure). Little’s MCAR test was used to evaluate whether data were missing completely at random, and the results indicated that there was no systematic pattern of missingness [χ2(324) = 354.51, *p* = 0.117].

For this study, 1,002 adolescents (82.5%) had data at age 13, 953 (78.4%) at age 15, and 929 (76.5%) at age 16. Overall, 70.3% of adolescents (*n* = 854) had data for at least one study variable at all three time points (ages 13, 15, and 16), while 29.7% (*n* = 361) had missing data at one or more of the time points. Adolescents who had data for all three assessments did not differ from those who did not have data in one or more assessments (*n* = 361) with respect to adolescent gender (*χ*^2^ (1) = 2.10, *p* = 0.149) or their country group (χ2 (1) = 1.28, *p* = 0.285). Additionally, MANOVAs revealed no difference between these two groups in terms of mother education and father education (Wilks’ λ = 1.00, *F*(2, 1035) = 0.99, *p* = 0.372), study variables at age 13 (Wilks’ λ = 0.99, *F*(7, 984) = 1.33, *p* = 0.234), or study variables at age 15 (Wilks’ λ = 0.99, *F*(7, 922) = 1.21, *p* = 0.293), and age 16 (Wilks’ λ = 1.00, *F*(4, 917) = 1.00, *p* = 0.407).

### Analysis Plan

First, preliminary analyses were conducted to examine descriptive statistics and zero-order bivariate correlations among study variables. Path analyses with observed scores were then performed using the *lavaan* package (Rosseel, 2012) and R software (R Core Team, 2016) to test the longitudinal associations among study variables. When testing the hypothesized model, missing values were estimated using full information maximum likelihood estimation (FIML), which allows for the inclusion of all available data points without imputing missing values. FIML provides unbiased parameter estimates under the assumption that data are Missing Completely at Random (MCAR) or Missing at Random (MAR) (Enders, 2010). Parameter estimates were calculated using maximum likelihood estimation with robust standard errors (MLR) to account for non-normality. However, when testing the indirect effects, maximum likelihood estimation (ML) was used, as this estimator is required for bootstrapping. Bootstrapping with 1,000 sampling was used to generate confidence intervals for the indirect effects.

**Fig. 1:**
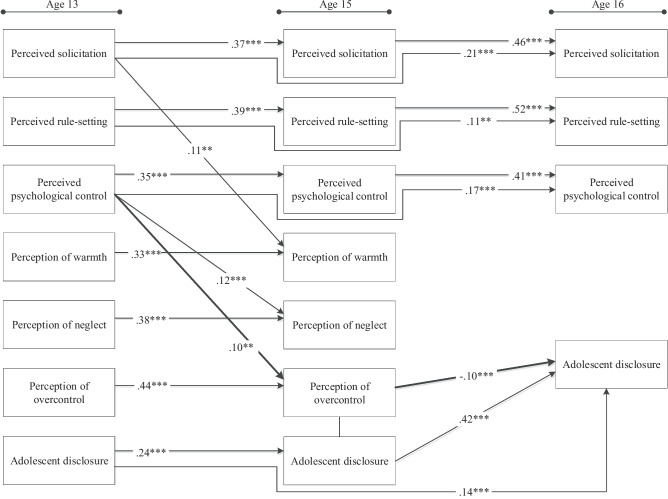
The Results of the Path Model for the Overall Sample. Standardized path coefficients are presented. Bolded lines indicate the mediated path from psychological control to disclosure. Nonsignificant paths (*p* > 0.05), covariates, and covariances are not displayed in the figure for simplicity. ** *p* < 0.01, *** *p* < 0.001

The model was first tested for the full sample and then multi-group path analysis was performed to examine whether there were differences in the hypothesized associations across the two cultural orientations. The analysis began with a model in which all paths were constrained to be equal across groups. This constrained model was then compared to a series of models in which sets of parameters were sequentially freed (paths from covariates, covariances among study variables, stability paths, and regressions among study variables), and evaluated whether model fit improved after each step. This iterative constraint-release approach allowed for testing whether the structural relations among study variables differed significantly across groups. In the models, between adjacent time points, the direct paths from perceived solicitation, rule-setting, and psychological control to the perceptions of warmth, neglect, and overcontrol (e.g., from perceived solicitation at age 13 to perception of overcontrol at age 15), and adolescent disclosure, and from the perceptions of warmth, neglect, and overcontrol to disclosure were included. Stability paths among the same variables were also estimated (e.g., from perceived solicitation at age 13 to perceived solicitation at ages 15 and 16; see Fig. 1). Adolescent gender and parental education (years of education of the most educated parent) were included as covariates. In addition, all variables measured in the same year were allowed to covary. Standardized scores (z-scores) were used in all models.

Model fit was evaluated based on three fit indices: the comparative fit index (CFI), the standardized root mean residual (SRMR), and the root mean square error of approximation (RMSEA) with confidence intervals. A value of CFI greater than 0.95 was considered as a good fit, and a value greater than 0.90 was considered as an acceptable fit of the model. For SRMR and RMSEA, a value less than 0.05 was considered as a good fit, and a value less than or equal to 0.08 was considered as an acceptable fit of the model (Browne & Cudeck, 1993; Hu & Bentler, 1999). To compare the fit of the nested models, Chen’s (2007) cutoff criteria for CFI and RMSEA differences were applied because the χ^2^ difference test is sensitive to sample size. A value of ΔCFI lower than −0.01 supplemented by a value of ΔRMSEA lower than 0.015 indicates invariance across groups for sample sizes greater than 300 (Chen, 2007). In addition, to examine whether the strength of indirect associations differed across cultural orientations, Wald tests of parameter constraints were conducted.

Finally, two sensitivity analyses were performed to evaluate the robustness of the current findings. First, the hypothesized model was re-estimated for the full sample using only the three disclosure items that specifically captured voluntary sharing of information, excluding the two secrecy/concealment items. Second, the main analysis was repeated after excluding participants with more than 20% missing data. These analyses allowed an assessment of whether the main conclusions were consistent when focusing on a narrower operationalization of disclosure and when reducing the impact of missing data.

## Results

### Preliminary Analyses

Descriptive statistics and zero-order bivariate correlations for the full sample are displayed in Table 1 (see Table S1a and S1b in the supplementary material for the corresponding values calculated separately for more individualistic and more collectivistic countries). In general, the pattern of correlations was similar across the two groups of countries except for a few differences (see bold values in Tables S1a and S1b for all differences). The negative correlation between perceived psychological control at age 13 and perception of warmth at age 15, the positive correlation between perceived rule-setting at age 13 and perception of overcontrol at age 15, and the negative correlation between perceived psychological control at age 15 and disclosure at age 16 were significant only in more individualistic countries. Perception of overcontrol at age 13 was positively and significantly correlated with disclosure at age 15 only in more collectivistic countries. There were also some differences between the two country groups in the correlations of perceived psychological control with solicitation and rule-setting. In more individualistic countries, perceived psychological control was negatively correlated with perceived solicitation at ages 13 and 16 (but not at age 15), whereas no significant correlations were found at any age in more collectivistic countries. In individualistic countries, perceived psychological control was positively correlated with perceived rule-setting at ages 15 and 16 (but not at age 13), while again, no significant correlations were observed in collectivistic countries. In both country groups, perceived solicitation and rule-setting were positively correlated at all assessment points. Adolescent gender and parental education were significantly correlated with some of the study variables (see Table S7 in the supplementary material for correlations by cultural orientation), so the two variables were included as covariates in the main analyses.

**Table 1:** Descriptive Statistics and Zero-order Bivariate Correlations of the Study Variables for the Overall Sample

Variable	1	2	3	4	5	6	7	8	9	M	SD	α	ωₜ
1. Age 13 Psychological Control	−									1.97	0.70	0.63	0.63
2. Age 13 Solicitation	−0.04	−								1.79	0.69	0.79	0.79
3. Age 13 Rule-setting	−0.02	0.43**	−							2.30	0.60	0.76	0.77
4. Age 13 Perception of Warmth	−0.18**	0.29**	0.22**	−						3.53	0.49	0.86/0.82^a^	0.87/0.83^a^
5. Age 13 Perception of Neglect	0.30**	−0.18**	−0.15**	−0.55**	−					1.48	0.46	0.72/0.71^a^	0.73/0.71^a^
6. Age 13 Perception of Overcontrol	0.27**	0.07*	0.15**	0.11**	0.10**	−				2.79	0.62	0.54/0.45^a^	0.54/46^a^
7. Age 13 Disclosure	−0.18**	0.44**	0.35**	0.46**	−0.31**	0.05	−			2.07	0.61	0.65	0.66
8. Age 15 Psychological Control	0.37**	0.03	0.06	−0.17**	0.26**	0.16**	−0.11**	−		1.97	0.72	0.63	0.65
9. Age 15 Solicitation	−0.07*	0.40**	0.18**	0.20**	−0.15**	0.08*	0.27**	−0.05	−	1.78	0.68	0.79	0.79
10. Age 15 Rule-setting	0.00	0.26**	0.41**	0.21**	−0.12**	0.19**	0.21**	0.12**	0.40**	2.17	0.66	0.76	0.82
11. Age 15 Perception of Warmth	−0.13**	0.20**	0.09**	0.45**	−0.32**	0.04	0.25**	−0.29**	0.41**	3.38	0.56	0.91/0.86^a^	0.91/0.87^a^
12. Age 15 Perception of Neglect	0.24**	−0.11**	−0.04	−0.36**	0.49**	−0.01	−0.20**	0.34**	−0.35**	1.55	0.46	0.76/0.70^a^	0.77/0.71^a^
13. Age 15 Perception of Overcontrol	0.24**	0.08*	0.12**	−0.03	0.14**	0.49**	0.04	0.34**	0.04	2.54	0.71	0.64/0.57^a^	0.64/0.57
14. Age 15 Disclosure	−0.12**	0.21**	0.14**	0.28**	−0.21**	0.05	0.36**	−0.20**	0.46**	2.02	0.61	0.66	0.67
15. Age 16 Psychological Control	0.33**	0.01	0.02	−0.16**	0.25**	0.21**	−0.11**	0.49**	−0.01	2.01	0.71	0.65	0.65
16. Age 16 Solicitation	−0.06	0.41**	20**	0.25**	−0.17**	0.06	0.27**	−0.03	0.56**	1.65	0.68	0.80	0.81
17. Age 16 Rule-setting	0.08*	0.22**	0.34**	0.15**	−0.07*	0.17**	0.19**	0.12**	0.29**	2.02	0.74	0.85	0.86
18. Age 16 Disclosure	−0.07*	0.21**	0.13**	0.25**	−0.20**	−0.01	0.33**	−0.12**	0.31**	1.96	0.59	0.67	0.67
	10	11	12	13	14	15	16	17					
11. Age 15 Perception of Warmth	0.19**												
12. Age 15 Perception of Neglect	−0.13**	−0.64**											
13. Age 15 Perception of Overcontrol	0.20**	0.04	0.16**										
14. Age 15 Disclosure	0.32**	0.46**	−0.38**	−0.04									
15. Age 16 Psychological Control	0.04	−0.13**	0.26**	0.33**	−0.09**								
16. Age 16 Solicitation	0.26**	0.36**	−0.26**	0.10**	0.39**	−0.08*							
17. Age 16 Rule-setting	0.57**	0.17**	−0.09**	0.22**	0.23**	0.12**	0.43**						
18. Age 16 Disclosure	0.18**	0.30**	−0.27**	−0.10**	0.56**	−0.18**	0.47**	0.31**					

^a^ α or ωₜ for adolescent report for fathers/α or ωₜ for adolescent report for mothers. * *p* < 0.05, ** *p* < 0.01

### Main Analyses

Path analysis in the full sample yielded an adequate fit to the data, S-Bχ^2^ (69) = 249.23, *p* < 0.001, CFI = 0.966, SRMR = 0.051, RMSEA = 0.046 (90-CI: 0.040 - 0.052). As shown in Fig. 1, (1) perceived solicitation at age 13 was positively related to perceptions of warmth at age 15; (2) perceived psychological control at age 13 was positively related to perceptions of neglect and perceptions of overcontrol at age 15; (3) perceptions of overcontrol at age 15 were negatively linked with disclosure at age 16. The tests of indirect effects revealed one significant indirect and negative link between psychological control at age 13 and disclosure at age 16 via perceptions of overcontrol at age 15, *β* = –0.010, *SE* = 0.004, 95% CI [–0.019, –0.003], *p* = 0.013 (see supplementary material – Table S8 for all path coefficients for the full sample including nonsignificant ones, Table S9 for path coefficients between covariates and study variables and Table S10 for covariances among study variables).

Multi-group path models were tested to evaluate potential differences across the country groups in these relations. The fully constrained model showed acceptable fit, S-Bχ² (258) = 590.58, p < 0.001, CFI = 0.939, SRMR = 0.068, RMSEA = 0.046 (90% CI [0.041, 0.051]). Releasing the paths from covariates to study variables at once, S-Bχ² (222) = 510.87, p < 0.001, CFI = 0.947, SRMR = 0.064, RMSEA = 0.046 (90% CI [0.041, 0.051]), did not improve fit (ΔRMSEA = 0.000, ΔCFI = 0.008), so constraints on paths from covariates were retained. Releasing all covariances between study variables at once, S-Bχ² (210) = 461.02, p < 0.001, CFI = 0.954, SRMR = 0.061, RMSEA = 0.044 (90% CI [0.039, 0.050]), likewise yielded no improvement (ΔRMSEA = –0.002, ΔCFI = 0.015), so constraints on covariances were retained. Releasing the stability paths at once, S-Bχ² (243) = 552.17, p < 0.001, CFI = 0.943, SRMR = 0.067, RMSEA = 0.046 (90% CI [0.041, 0.051]), also showed no better fit (ΔRMSEA = 0.000, ΔCFI = 0.004), so constraints on stability paths were retained. Finally, releasing all regressions among the study variables at once, S-Bχ² (237) = 569.85, p < 0.001, CFI = 0.939, SRMR = 0.067, RMSEA = 0.048 (90% CI [0.043, 0.053]), did not improve fit (ΔRMSEA = 0.002, ΔCFI = 0.000). Taken together, the series of model comparisons indicated no differences between these nested models, meaning that relations among study variables did not differ significantly across more individualistic and more collectivistic countries.

Wald tests indicated no differences in the strength of indirect relations between more individualistic and more collectivistic countries (all *p*s > 0.05). In sum, even though the hypothesized associations among study variables were supported to some extent, no evidence was found for country group differences proposed in the hypotheses.

### Sensitivity Analyses

A sensitivity analysis by re-estimating the hypothesized model for the full sample using only the three items specifically assessing voluntary disclosure (i.e., excluding the two secrecy/concealment items) revealed that the model fit was acceptable, S-Bχ^2^ (69) = 242.68, *p* < 0.001, CFI = 0.968, SRMR = 0.050, RMSEA = 0.046 (90-CI: 0.040 - 0.052). The overall pattern of results was highly consistent with the main analysis in terms of direction and significance of the effects, including the indirect effect. Notably, two additional significant paths emerged: solicitation at age 13 positively predicted disclosure at age 15 (*β* = 0.11, *p* = 0.006), and solicitation at age 15 positively predicted disclosure at age 16 (*β* = 0.09, *p* = 0.012). These findings suggest that while the main conclusions are robust, focusing exclusively on disclosure items underscores stronger associations between solicitation and disclosure.

An additional sensitivity analysis on the overall sample, excluding 312 participants with more than 20% missing data (*n* = 903) yielded an acceptable model fit: S-Bχ²(69) = 247.94, *p* < 0.001; CFI = 0.967; SRMR = 0.053; RMSEA = 0.054 (90% CI: 0.047–0.061). The pattern of autoregressive and cross-lagged associations remained highly consistent with the main analyses, with all paths showing similar significance and direction. These results indicate that current findings are robust to the handling of missing data.

## Discussion

Past studies have offered important insights into the links between parenting and adolescent disclosure, but they often examined autonomy-related parenting practices in isolation or relied on cross-sectional designs (e.g., Baudat et al., 2020; Keijsers et al., 2010), which limits understanding of how these parenting practices uniquely shape adolescent disclosure over time. In addition, little is known whether these associations differ across cultural contexts. By simultaneously modeling three autonomy-relevant parenting practices—while controlling for one another and accounting for their temporal stability and concurrent associations—this study offers a clearer and developmentally sensitive examination of adolescent disclosure. Grounded in Self-Determination Theory (Deci & Ryan, 2000), the study aimed to examine how the three parenting practices contribute differentially to adolescents’ willingness to share information with their parents. Moreover, by testing mediational pathways involving adolescents’ perceptions of parental warmth, neglect, and overcontrol, the current findings offer a theoretically grounded explanation for how parenting can foster or hinder disclosure. This mediational focus responds to theoretical and empirical suggestions that adolescents’ interpretations of parenting practices play a critical role in how those behaviors affect the parent–child relationship (Soenens & Vansteenkiste, 2020). Finally, by addressing these questions within a cross-cultural framework (i.e., by testing whether these associations varied across more individualistic and more collectivistic countries), the study contributes to a broader understanding of the role of culture in the disclosure dynamics during adolescence. As outlined below, the results revealed differential relations between the three perceived parenting practices with adolescent disclosure and adolescents’ perceptions of warmth, neglect, or overcontrol over time, after controlling for stability and concurrent relations. Contrary to expectations, these associations did not differ across more individualistic and more collectivistic countries.

### Psychological Control and Disclosure

Of the three autonomy-related parenting practices assessed, the only one that predicted adolescent disclosure over time when all three were simultaneously considered was perceived psychological control. The direct cross-lagged relationships between perceived psychological control and disclosure between adjacent time-points were not statistically significant but, consistent with the assumptions of Self-Determination Theory and Hypothesis 3, perceived psychological control at age 13 predicted lower adolescent disclosure at age 16 via increased perception of overcontrol at age 15. This was true across country groups. A previous study with a shorter time interval (7 months) between the two waves did not reveal a longitudinal direct link between psychological control and adolescent disclosure after statistically controlling for behavioral control and parental warmth although their concurrent associations were negative and significant (Kearney & Bussey, 2015). However, another longitudinal study that did not statistically control for other aspects of autonomy-related parenting revealed that both maternal and paternal psychological control were related to less disclosure about personal issues (e.g., free time activities) via lower perceived parental support. In addition, more maternal psychological control directly predicted less disclosure about personal issues one year later when mediating variables (parental support and negative interactions with parent) were not included (Song & Smetana, 2024). Therefore, it seems that when other aspects of autonomy-related parenting such as monitoring are taken into account, then psychological control’s link with adolescent disclosure over time gets weaker. However, the current results suggest that it is still indirectly and uniquely related to less adolescent disclosure over time via its positive association with perceptions of overcontrol.

The current findings are consistent with theory indicating that psychological control is a critical parenting practice with respect to autonomy development (Soenens & Vansteenkiste, 2010), including that it is a relatively important predictor of declines in disclosure from early to middle adolescence. The present findings further show that adolescents’ perceptions of overcontrol could account for this phenomenon across groups of countries that differ in average individualistic or collectivistic cultural orientations. Previous research indicates that believing an issue is not under parents’ jurisdiction is among the reasons for adolescent nondisclosure (Darling et al., 2006). The current results are consistent with this in that when adolescents feel that their parents want too much control, adolescents voluntarily talk less about their daily activities, perhaps as a way of setting boundaries and protecting their privacy. According to Self-Determination Theory, adolescents’ need for autonomy may be undermined by psychological control across cultural contexts (Soenens & Vansteenkiste, 2010). This theoretical perspective might help explain why, in the present study, only psychological control longitudinally predicted disclosure via perceptions of overcontrol.

### Solicitation and Disclosure

Why were the longitudinal associations between solicitation or rule-setting and adolescent disclosure that were expected in Hypotheses 1 and 2, not observed? Previous research has provided mixed results regarding the relation of solicitation with adolescent disclosure (e.g., Baudat et al., 2020; Hamza & Willoughby, 2011; Kerr et al., 2010). The present study sought to shed light on the inconsistency by including several countries that varied in degree of individualistic and collectivistic orientation, by examining different parenting practices controlling for one another, and by examining possible mechanisms underlying the hypothesized associations. In line with most of previous cross-sectional studies (e.g., Baudat et al., 2020; Stattin & Kerr, 2000), solicitation was found to be positively and significantly associated with disclosure concurrently. However, after controlling for perceived psychological control and rule-setting, the cross-lagged direct and indirect associations (i.e., via their relations of perceptions of warmth, neglect, and overcontrol) between perceived solicitation and adolescent disclosure were insignificant. In other words, perceived solicitation did not account for a unique proportion of variance in disclosure after statistically controlling for other autonomy-relevant parenting practices and previous levels of disclosure.

Although this finding contrasts with a few studies that reported a positive and significant cross-lagged association between perceived solicitation and adolescent disclosure (e.g., Hamza & Willoughby, 2011), it aligns with other longitudinal research that found no evidence that solicitation directly or indirectly promotes disclosure over time (e.g., Kerr et al., 2010; Tilton-Weaver, 2014). It may be that attempts to obtain information beyond what they can obtain via disclosure do not lead to greater disclosure over time, at least from early to middle adolescence. Alternatively, current results might suggest that solicitation has temporally proximal associations with disclosure rather than long-term associations, especially when other parenting practices are accounted for. Given the possibility that the actual relationships between these variables would be concurrent and difficult to detect after adjusting for within-wave associations and stability among the same variables, current results do not necessarily imply that parental solicitation is an ineffective strategy for eliciting disclosure. Indeed, a diary study (Solís et al., 2015), and a study with weekly assessments (Keijsers et al., 2016) suggest that adolescents disclose more when their parents solicit information, a finding which may not appear in longitudinal studies.

Importantly, the sensitivity analysis that excluded secrecy/concealment items yielded a somewhat different picture: perceived solicitation at age 13 predicted disclosure at age 15, and perceived solicitation at age 15 predicted disclosure at age 16. This suggests that the lack of longitudinal effects in the main analyses might partly reflect the inclusion of items capturing secrecy, which could have diluted the predictive role of solicitation. Thus, solicitation appears to be more consistently linked to later disclosure when disclosure is operationalized more narrowly. The current results, therefore, underscore that both operationalization choices and measurement content matter for interpreting longitudinal associations.

### Rule-Setting and Disclosure

Prior research has generally shown nonsignificant or negative associations with adolescent disclosure (e.g., Darling et al., 2009; Kapetanovic et al., 2019; Kerr et al., 2010). The current study aimed to extend existing knowledge by examining whether rule-setting accounts for unique variance in adolescent disclosure over time through its associations with adolescents’ perceptions of parental warmth, neglect, and overcontrol and after controlling for both perceived solicitation and psychological control. Additionally, the study investigated whether the association between rule-setting and disclosure varies across countries differing in their levels of individualistic versus collectivistic cultural orientation.

Perceived rule-setting did not directly or indirectly predict disclosure over time even though their concurrent relations with each other were positive and significant. In general, available longitudinal studies have shown nonsignificant correlation between parental rule-setting and disclosure after statistically controlling for parental solicitation and previous levels of disclosure (e.g., Kerr et al., 2010; Willoughby & Hamza, 2011). Accordingly, current findings are consistent with earlier research in showing that having expectations and rules about adolescents’ activities are less likely to elicit increased disclosure over time than other parenting practices. It is also important to note that both perceived solicitation and rule-setting did not predict less disclosure or higher perceptions of overcontrol over time after controlling for perceived psychological control, which does not support the idea that they might backfire, even in more individualistic countries, when they are not displayed in an intrusive manner (see Rodríguez-Meirinhos et al., 2020). Moreover, the sensitivity analysis indicated that solicitation might, under a narrower operationalization of disclosure (excluding secrecy items), even predict greater disclosure across waves. This suggests that solicitation is unlikely to undermine disclosure and, in some cases, might play a modest promotive role depending on how disclosure is measured.

### Distinct Associations with Perceptions of Warmth, Neglect, and Overcontrol

Previous work suggests that interpretations of parental intent might differ for the different autonomy-relevant practices, and consequently they may have differential influence on adolescent-parent child relationship (Hawk et al., 2009; Selçuk et al., 2022; Soenens & Vansteenkiste, 2020). Therefore, it was expected adolescents’ perceptions of warmth, neglect, and overcontrol would mediate the link between the relations of three parenting practices and adolescent disclosure. Although current results did not support the expected mediating relationships stated in Hypotheses 1 to 3 (except the mediating role of perception of overcontrol for psychological control), distinct patterns emerged regarding the relations of three parenting practices with adolescents’ perceptions of parental warmth, neglect, and overcontrol.

It is interesting that adolescents feel both more neglected and more overcontrolled in response to psychological control, because these constructs imply a potential desire for both more and less parental attention. However, the finding makes sense if one considers that psychologically controlling parents tend to withdraw important positive emotions from their children while they also intrude on and attempt to manipulate their children’s feelings and thoughts, as well as behaviors. This finding supports Self-Determination Theory and other (e.g., Kagitcibasi, 2005) perspectives proposing that psychological control undermines adolescents’ needs for autonomy, competence, and relatedness (Soenens & Vanstenkiste, 2010). Although the accumulated evidence shows that adolescents attribute negative meanings to psychological control practices regardless of cultural context, some past findings (Chen et al., 2016; Helwig et al., 2014) have suggested that adolescents from more individualistic countries or cultural groups have especially negative perceptions regarding certain aspects of psychological control (e.g., guilt induction). Such differences were not detected in the present study. If the focus had been placed on those aspects of psychological control that are more likely to be interpreted differently depending on cultural orientation, such differences might have been observed. Taken together, in line with previous findings (e.g., Urry et al., 2011), the current findings suggest that psychological control might undermine the adolescent-parent relationship (i.e., through adolescents feeling neglected or overcontrolled).

Although more perceived parental solicitation at age 13 did not predict later adolescent disclosure (or overcontrol) over time, it did predict higher perceptions of warmth at age 15 across the two country groups. This finding suggests that when parents ask about adolescents’ friends, interests, and activities - controlling for their use of rule-setting or psychological control – adolescents may interpret this as an expression of care and affection, reinforcing the idea that parental solicitation can foster feelings of closeness and warmth (Fernandez et al., 2021; Garthe et al., 2018), just as showing interest through questions strengthens bonds between acquaintances, friends, and other family members. Supporting this argument, a focus group study with Croatian adolescents revealed that adolescents tended to evaluate unobtrusive open-ended questions such as “How was your day at school?” as an indication of parental interest and offering help, and from adolescents’ perspective, such questions facilitated disclosure (Tokić & Pećnik, 2011).

Unexpectedly, adolescents who perceived more parental warmth were not more inclined, a year later, to share information with their parents. Although this finding contrasts with other research indicating that parental warmth and responsiveness is an important and consistent predictor of adolescent disclosure (Liu et al., 2020; Villarreal & Nelson, 2022), current results might imply that perceived overcontrol has a more critical role than perceived warmth, by discouraging disclosure. It also implies that parental solicitation does not guarantee increased adolescent disclosure over time because of its association with perceptions of warmth; rather adolescent disclosure is predicted by the overall context of several parenting practices.

Given that no significant longitudinal link was found between perceived solicitation at age 13 and feeling overcontrolled at age 15, the results might appear to conflict with those from past studies conducted in countries that have a more individualistic orientation such as Sweden and the Netherlands (Hawk et al., 2016; Kapetanovic et al., 2020) and one study in China (although it was a modest association), which is more collectivistic (Hawk, 2017), which found significant associations between solicitation and perceptions of privacy invasion or feeling overly controlled. Perhaps their results differed because they did not control for psychological control practices, and they were cross-sectional studies. In fact, in the present study, the zero-order bivariate correlation between perceived solicitation and perception of overcontrol at age 13 (but not at age 15) was positive and significant in more individualistic but not in more collectivistic countries (see Tables S1 and S2). Yet, in multivariate analyses controlling for perceived rule-setting and psychological control, and when perceptions of warmth, neglect, and overcontrol were considered together as possible outcomes, no evidence was found suggesting that adolescents interpret solicitation negatively in either cultural context. Overall, the findings suggest that solicitation apart from psychological control does not have the same worrisome repercussions.

Unlike perceived solicitation, perceived rule-setting at age 13 was not significantly related to perceptions of warmth at age 15. This finding suggests that solicitation and rule-setting, apart from each other, have differential associations with parent-adolescent relationship over time. In addition, even though the zero-order bivariate correlation between perceived rule-setting and perception of overcontrol at ages 13 and 15 were positive and significant in more individualistic but not in more collectivistic countries (see Tables S1 and S2 in the supplementary material), no evidence was found that adolescents feel more overcontrolled over time in response to rule-setting. Although this finding contradicts prior cross-sectional studies (e.g., Kapetanovic et al., 2020), it is in line with a previous longitudinal study which found no longitudinal association between rule-setting and increased feelings of being overcontrolled after accounting for psychologically controlling and restrictive parenting behaviors (Kakihara et al., 2010). Together with this earlier finding, the present findings could imply that perceived rule-setting – apart from the other practices measured – does not lead adolescents to believe that their parents are acting in excessively controlling ways over time. One factor that might help explain these findings is parental authority legitimacy—that is, the extent to which adolescents believe parents have the right to monitor or restrict their behavior across different life domains, such as leisure activities (Darling et al., 2005; Keijsers & Laird, 2014; Smetana et al., 2015). When legitimacy is granted, even frequent solicitation or rule-setting might be interpreted as caring and normative, whereas the same behaviors might be experienced as intrusive or controlling if legitimacy is not recognized. Perceptions of legitimacy vary across cultural contexts (Darling et al., 2005; Lansford, 2022). It is important to note that although overcontrol might be related to the broader concept of parental authority legitimacy (i.e., beliefs about the appropriate boundaries of parental authority; Smetana et al., 2015), the current study did not explicitly assess legitimacy perceptions. Rather, the focus was placed on adolescents’ perceptions of being overly controlled. Incorporating legitimacy perceptions in future research might therefore help explain cultural variation in how autonomy-relevant parenting practices relate to perceptions of warmth, neglect, and overcontrol as well as adolescent disclosure. Alternatively, considering that no significant cross-lagged associations were found between perceived solicitation or rule-setting at age 13 and perceived neglect at age 15, perhaps the length of the lag (two years) prevented identifying more short-term cross-lagged relationships between these variables.

### Limitations and Future Directions

The strengths of the current study include a relatively large number of participants from eight countries, longitudinal data, and analyses that predict change in adolescent disclosure (i.e., controlling for earlier levels of disclosure). There are also some limitations that need to be recognized. First, this was a secondary analysis, and direct measures of adolescents’ feelings and perceptions in response to specific parental behaviors were not available. Adolescents’ ratings on the subscales of the Parental Acceptance-Rejection/Control Questionnaire-Short Form (PARQ/Control-SF; Rohner, 2005) measure adolescents’ general perceptions of warmth, neglect, and especially high levels of control. In the context of this longitudinal study, these measures allowed examination of how perceived solicitation, rule-setting, and psychological control predicted the development of perceived warmth, neglect, or overcontrol in the parent-child relationship. Empirically, there is reason to believe these measures are indicators of the constructs of interest. For instance, parental warmth evaluated with the same subscale was strongly related to perceived mattering to parents (r = 0.72 for mothers and 0.69 for fathers; Marshall, 2001). However, to directly test differences in adolescents’ interpretations of parenting practices across more individualistic and collectivistic countries, future studies should directly assess adolescents’ interpretations of specific parenting practices.

Second, as alluded to earlier, it is an oversimplification to categorize countries as more individualistic versus more collectivistic. There is variability within each country in terms of individualism and collectivism, meaning that some participants do not conform to individualism-collectivism distinction. Therefore, the absence of predicted country group differences in the current study might be because actual differences of importance may not have been captured. Future studies could assess individualism and collectivism at the individual level, as well as account for other cultural dimensions and country-level differences such as economic growth and industrialization level.

Third, it should also be noted that the indirect effect (i.e., between perceived psychological control and disclosure via perception of overcontrol) that was found to be significant was small in size. However, it might be still important evidence given that it was tested over three years with a conservative model that includes stability and concurrent associations between study variables.

Fourth, only adolescent reports were used in the present study, so shared method variance might have affected the magnitude of the relations among study variables (Podsakoff et al., 2012). Although it is adolescents’ perceptions that are likely to matter in these associations, understanding how actual parental behaviors relate to adolescents’ perceptions of those behaviors is important. Moreover, hypothesized associations were tested between early and middle adolescence. Future studies should examine how the associations may vary across different stages of adolescence.

Fifth, there are some limitations in the content of the measures. The measure of disclosure did not differentiate between disclosure and secrecy. As these two constructs are inversely but moderately related to each other (Smetana et al., 2006), future research might study whether this distinction matters with respect to the predictors examined. It is also worth noting, however, that the main conclusions were robust across sensitivity analyses. When the secrecy/concealment items were excluded and the focus was placed only on voluntary disclosure, solicitation more consistently predicted disclosure across waves, but the broader pattern of indirect and direct effects remained unchanged. In addition, given prior work indicating that disclosure might vary by type (e.g., routine vs. self-disclosure; Tilton-Weaver et al., 2014) and by domain (e.g., personal activities, issues concerning the safety and health of the adolescent; Song & Smetana, 2024), future studies should investigate whether the associations observed here generalize across different aspects of disclosure.

Sixth, the relatively low reliability of some measures— the perception of overcontrol, especially in collectivistic countries at age 13, and psychological control—should be acknowledged. Although McDonald’s omega and item–total correlations supported modest but acceptable internal consistency for short three-item scales, caution is warranted in interpreting results for constructs with lower reliability. Nonetheless, several considerations help mitigate these concerns: the longitudinal design reduced the likelihood that small reliability issues would drive effects, and tests of metric invariance ensured comparable item functioning across cultural contexts.

Seventh, the current study did not differentiate between perceptions of mothers’ and fathers’ behavior in this study or take into account other aspects of family structure. Given that the effects of parenting practices may differ as a function of parent gender (e.g., Bean et al., 2003), and that fathers – especially from more collectivistic cultures – might have more distant relationships with their children compared to mothers (Alampay, 2014), examining adolescents’ perceptions regarding paternal and maternal practices separately across countries might be a crucial direction for future research. In addition, aspects of family structure such as whether families include two parents, multiple siblings, or grandparents or other extended family members in the household might be related to parenting and adolescents’ disclosure, an important direction for future research. Moreover, adolescent gender was not explicitly tested as a potential moderator of the observed associations. Although gender was included as a covariate, exploring how parenting practices might differentially relate to adolescent disclosure based on gender could be a meaningful direction for future work. Gender differences might vary across cultural contexts, and examining gender as a moderator could yield important insights into how parenting is associated with disclosure in different subgroups. It is recommended that future studies test gender as a moderator alongside other cultural variables to better understand these dynamics. In addition, because families were recruited from mainly urban settings, the findings might not generalize to other types of settings (e.g., rural communities).

Eighth, although Little’s MCAR test suggested no systematic pattern of missingness, the relatively high proportion of missing data in some variables might still have influenced the findings. Thus, the results should be interpreted with appropriate caution.

Finally, several methodological considerations regarding the statistical approach should be noted. Although the models accounted for longitudinal associations, they did not differentiate within- versus between-person variation. Standard cross-lagged panel models aggregate these effects, meaning that some observed or nonsignificant findings may reflect between-person differences rather than within-person processes over time. Future studies could apply random-intercept cross-lagged panel models (RI-CLPM; Hamaker et al., 2015; Keijsers, 2016) to more precisely disentangle these processes. Such approaches may help clarify some of the discrepancies observed with previous cross-sectional and longitudinal studies and provide a more fine-grained understanding of how changes in parenting practices relate to adolescent disclosure over time.

## Conclusion

Prior research has examined associations between parental solicitation, rule-setting, and psychological control—and adolescent disclosure, yet these practices have rarely been examined simultaneously, studies have often relied on cross-sectional designs, and research has largely been limited to single-country contexts. These limitations constrain insight into their unique associations across time and potential differences across cultural contexts. The current study addressed these gaps by simultaneously examining solicitation, rule-setting, and psychological control in a longitudinal design, testing their unique associations with adolescent disclosure through adolescents’ perceptions of parental warmth, neglect, and overcontrol across more individualistic and more collectivistic countries. Results of this study suggest that in both more individualistic and more collectivistic countries, solicitation is linked with an increased perception of parental warmth. Practical implications of these findings are that there might be danger in *not asking* questions as adolescents might be less likely to feel loved over time if they do not think their parents are interested in their daily activities and genuinely care about their well-being. Additionally, it seems that from the adolescent perspective, neither solicitation nor rule-setting ensures increased adolescent disclosure over time. However, the sensitivity analysis revealed that solicitation might predict greater disclosure when disclosure is operationalized more narrowly, suggesting that its role in fostering openness might depend on how disclosure is measured. Furthermore, psychological control seems to be associated with decreased disclosure partly because adolescents with psychologically controlling parents are likely to think that their parents exert excessive control on their life. Therefore, although solicitation and rule-setting do not interfere with disclosure and might have other benefits, it seems that parents are most likely to enhance the likelihood of adolescent disclosure by refraining from psychological control practices. Together, these findings advance understanding of adolescent disclosure by showing that different autonomy-relevant parenting practices are differentially associated with adolescents’ disclosure over time.

## Supplementary information

Supplementary Material

## Declarations

### Data Sharing Declaration

This manuscript’s data will not be deposited.
