# Supplementary material for: A Longitudinal Study of Parental Solicitation, Rule-Setting, and Psychological Control as Predictors of Adolescent Disclosure across More Individualistic and More Collectivistic Countries
Source: J Youth Adolesc. 2026 Jan 7;55(2):516–35. doi: 10.1007/s10964-025-02311-8 (PMC12894135; doi:10.1007/s10964-025-02311-8)
Supplement: Supplementary file 1 — Supplementary Material [file 10964_2025_2311_MOESM1_ESM.docx]

**Supplementary Material**

**Measurement Invariance**

For each scale, measurement invariance was tested using multi-group confirmatory factor analysis (CFA) to ensure that the scales evaluate the same constructs across more individualistic and more collectivistic countries. Using the MLR estimator, configural (i.e., all parameters were allowed to be free), metric (i.e., loadings were constrained to be equal across the groups), and scalar invariance (i.e., both loadings and intercepts were constrained to be equal across the groups) were tested, and the relative fit of these nested models was compared using Chen’s (2007) cutoff criteria. Results indicated at least metric invariance of the measures across the two cultural orientations (for details of all measurement invariance analyses, see Table S2 in this supplementary material).

**Bivariate Correlations**

In general, the pattern of correlations was similar across the two cultural orientations (see Table S1a and Table S1b).

**Supplemental References**

Chen, F. F. (2007). Sensitivity of goodness of fit indexes to lack of measurement invariance. *Structural Equation Modeling, 14*, 464–504. <https://doi.org/10.1080/10705510701301834>

**Table S1a**

*Descriptive Statistics and Zero-order Bivariate Correlations of the Study Variables for More Individualistic Countries*

| Variable | 1 | 2 | 3 | 4 | 5 | 6 | 7 | 8 | 9 | M | SD | α | ωₜ |
| --- | --- | --- | --- | --- | --- | --- | --- | --- | --- | --- | --- | --- | --- |
| 1. Age 13 Psychological Control | − |  |  |  |  |  |  |  |  | 1.83 | 0.64 | .66 | .66 |
| 2. Age 13 Solicitation | **-.14**** | − |  |  |  |  |  |  |  | 1.69 | 0.64 | .75 | .75 |
| 3. Age 13 Rule-setting | .02 | .45** | − |  |  |  |  |  |  | 2.26 | 0.60 | .76 | .77 |
| 4. Age 13 Perception of Warmth | -.21** | .37** | .17** | − |  |  |  |  |  | 3.61 | 0.45 | .88/.82^a^ | .88/.83^a^ |
| 5. Age 13 Perception of Neglect | .25** | -.29** | -.13** | -.58** | − |  |  |  |  | 1.40 | 0.40 | .73/.67^a^ | .74/67^a^ |
| 6. Age 13 Perception of Overcontrol | .31** | **.09*** | **.20**** | **.01** | **.08** | − |  |  |  | 2.77 | 0.63 | .60/.50^a^ | .63/.54^a^ |
| 7. Age 13 Disclosure | -.23** | .46** | .27** | .45** | -.32** | **.00** | − |  |  | 2.08 | 0.59 | .71 | .71 |
| 8. Age 15 Psychological Control | .37** | -.08 | .07 | -.20** | .20** | .18** | **-.16**** | − |  | 1.83 | 0.66 | .59 | .60 |
| 9. Age 15 Solicitation | **-.10*** | .46** | .19** | .23** | -.17** | .07 | .29** | -.06 | − | 1.75 | 0.65 | .77 | .78 |
| 10. Age 15 Rule-setting | .02 | .29** | .45** | .22** | -.19** | .23** | .22** | **.13**** | .41** | 2.08 | 0.68 | .81 | .82 |
| 11. Age 15 Perception of Warmth | **-.13**** | .26** | .10* | .46** | -.32** | **-.03** | .24** | -.34** | .38** | 3.43 | 0.53 | .92/.86^a^ | .92/86^a^ |
| 12. Age 15 Perception of Neglect | .16** | -.16** | -.06 | -.35** | .49** | -.03 | -.18** | .33** | -.29** | 1.49 | 0.44 | .78/.70^a^ | .79/.71^a^ |
| 13. Age 15 Perception of Overcontrol | .22** | .09 | **.17**** | -.06 | .10* | .54** | .02 | .33** | .02 | 2.50 | 0.73 | .70/.62^a^ | .71/.65^a^ |
| 14. Age 15 Disclosure | -.14** | .26** | .11* | .25** | -.26** | **-.01** | .40** | -.27** | .40** | 2.02 | 0.60 | .71 | .72 |
| 15. Age 16 Psychological Control | .32** | -.08 | .03 | -.17** | .19** | .21** | **-.16**** | .49** | -.11* | 1.90 | 0.66 | .63 | .64 |
| 16. Age 16 Solicitation | **-.14**** | .44** | 20** | .23** | -.17** | .05 | .26** | -.07 | .58** | 1.64 | 0.66 | .79 | .79 |
| 17. Age 16 Rule-setting | .07 | .19** | .34** | .10* | -.10* | **.22**** | .16** | **.12**** | .28** | 1.86 | 0.80 | .86 | .87 |
| 18. Age 16 Disclosure | -.07 | .21** | .11* | .23** | -.22** | .00 | .36** | **-.18**** | .29** | 1.95 | 0.60 | .71 | .72 |
|  |  |  |  |  |  |  |  |  |  |  |  |  |  |
|  | 10 | 11 | 12 | 13 | 14 | 15 | 16 | 17 |  |  |  |  |  |
| 11. Age 15 Perception of Warmth | .15** |  |  |  |  |  |  |  |  |  |  |  |  |
| 12. Age 15 Perception of Neglect | **-.07** | -.63** |  |  |  |  |  |  |  |  |  |  |  |
| 13. Age 15 Perception of Overcontrol | **.29**** | **-.03** | .12** |  |  |  |  |  |  |  |  |  |  |
| 14. Age 15 Disclosure | .23** | .41** | -.32** | **-.09*** |  |  |  |  |  |  |  |  |  |
| 15. Age 16 Psychological Control | .06 | **-.16**** | .23** | .31** | **-.18**** |  |  |  |  |  |  |  |  |
| 16. Age 16 Solicitation | .24** | .32** | -.23** | .08 | .35** | -.17** |  |  |  |  |  |  |  |
| 17. Age 16 Rule-setting | .59** | .13** | -.10* | .27** | **.18**** | .12** | .39** |  |  |  |  |  |  |
| 18. Age 16 Disclosure | .15** | .29** | -.25** | **-.11*** | .63** | -.25** | .41** | .24** |  |  |  |  |  |

*Note*. ^a^ α or ωₜ for adolescent report for fathers/α or ωₜ for adolescent report for mothers. * *p* < .05, ** *p* < .01. Correlation coefficients that are significant only in one group were presented in bold.

**Table S1b**

*Descriptive Statistics and Zero-order Bivariate Correlations of the Study Variables for More Collectivistic Countries*

| Variable | 1 | 2 | 3 | 4 | 5 | 6 | 7 | 8 | 9 | M | SD | α | ωₜ |
| --- | --- | --- | --- | --- | --- | --- | --- | --- | --- | --- | --- | --- | --- |
| 1. Age 13 Psychological Control | − |  |  |  |  |  |  |  |  | 2.12 | 0.73 | .59 | .59 |
| 2. Age 13 Solicitation | **-.03** | − |  |  |  |  |  |  |  | 1.91 | 0.74 | .82 | .81 |
| 3. Age 13 Rule-setting | -.08 | .41** | − |  |  |  |  |  |  | 2.35 | 0.60 | .75 | .76 |
| 4. Age 13 Perception of Warmth | -.11* | .29** | .30** | − |  |  |  |  |  | 3.45 | 0.52 | .85/.82^a^ | .86/.83^a^ |
| 5. Age 13 Perception of Neglect | .29** | -.16** | -.20** | -.51** | − |  |  |  |  | 1.58 | 0.50 | .71/.71^a^ | .71/.72^a^ |
| 6. Age 13 Perception of Overcontrol | .22** | **.03** | **.08** | **.23**** | **12**** | − |  |  |  | 2.81 | 0.61 | .50/.43^a^ | .55/.55^a^ |
| 7. Age 13 Disclosure | -.12** | .43** | .44** | .48** | -.31** | **.11*** | − |  |  | 2.07 | 0.64 | .62 | .63 |
| 8. Age 15 Psychological Control | .32** | .06 | .02 | -.10* | .26** | .11* | **-.09** | − |  | 2.14 | 0.76 | .67 | .67 |
| 9. Age 15 Solicitation | **-.07** | .35** | .15** | .19** | -.15** | .09 | .24** | -.06 | − | 1.81 | 0.71 | .80 | .80 |
| 10. Age 15 Rule-setting | -.08 | .18** | .35** | .26** | -.10* | .13** | .21** | **.05** | .39** | 2.28 | 0.63 | .79 | .81 |
| 11. Age 15 Perception of Warmth | **-.09** | .19** | .11* | .44** | -.31** | **.13**** | .27** | -.21** | .46** | 3.32 | 0.59 | .90/.87^a^ | .90/.87^a^ |
| 12. Age 15 Perception of Neglect | .28** | -.12** | -.05 | -.36** | .48** | .00 | -.24** | .32** | -.44** | 1.62 | 0.48 | .74/.70^a^ | .74/.70^a^ |
| 13. Age 15 Perception of Overcontrol | .24** | .05 | **.05** | .02 | .17** | .43** | .07 | .34** | .07 | 2.58 | 0.68 | .58/.54^a^ | .59/.56^a^ |
| 14. Age 15 Disclosure | -.11* | .18** | .17** | .32** | -.18** | **.12*** | .32** | -.13** | .54** | 2.02 | 0.62 | .62 | .65 |
| 15. Age 16 Psychological Control | .30** | .03 | -.03 | -.11* | .25** | .19** | **-.07** | .45** | -.11* | 2.14 | 0.75 | .65 | .65 |
| 16. Age 16 Solicitation | **.01** | .39** | 21** | .29** | -.18** | .08 | .28** | .01 | .53** | 1.67 | 0.69 | .81 | .82 |
| 17. Age 16 Rule-setting | .00 | .21** | .30** | .30* | -.14** | **.08** | .25** | **.01** | .32** | 2.20 | 0.63 | .81 | .82 |
| 18. Age 16 Disclosure | -.08 | .21** | .14* | .29** | -.19** | -.02 | .31** | **-.08** | .35** | 1.96 | 0.59 | .62 | .64 |
|  |  |  |  |  |  |  |  |  |  |  |  |  |  |
|  | 10 | 11 | 12 | 13 | 14 | 15 | 16 | 17 | 18 |  |  |  |  |
| 11. Age 15 Perception of Warmth | .27** | − |  |  |  |  |  |  |  |  |  |  |  |
| 12. Age 15 Perception of Neglect | **-.24**** | -.64** | − |  |  |  |  |  |  |  |  |  |  |
| 13. Age 15 Perception of Overcontrol | **.07** | **.14**** | .19** | − |  |  |  |  |  |  |  |  |  |
| 14. Age 15 Disclosure | .44** | .51** | -.45** | **.02** | − |  |  |  |  |  |  |  |  |
| 15. Age 16 Psychological Control | -.03 | **-.07** | .24** | .34** | **.00** | − |  |  |  |  |  |  |  |
| 16. Age 16 Solicitation | .28** | .41** | -.31** | .12* | .42** | .00 | − |  |  |  |  |  |  |
| 17. Age 16 Rule-setting | .52** | .29** | -.16** | .12* | .31** | **.05** | .50** | − |  |  |  |  |  |
| 18. Age 16 Disclosure | .22** | .33** | -.30** | **-.08** | .49** | -.12* | .55** | .44** | − |  |  |  |  |

*Note*. ^a^ α or ωₜ for adolescent report for fathers/α or ωₜ for adolescent report for mothers. * *p* < .05, ** *p* < .01. Correlation coefficients that are significant only in one group were presented in bold.

**Table S2**

*Results of Multiple Group Confirmatory Factor Analyses*

| **Measures** | **Models** | **df** | **S-Bχ2** | **CFI** | **SRMR** | **RMSEA (90-CI)** | Δ**RMSEA** | Δ**CFI** |
| --- | --- | --- | --- | --- | --- | --- | --- | --- |
| Age 13 Psychological Control | Configural Invariance | 0 | 0.00 | 1.00 | .000 | .000 (.000-.000) |  |  |
|  | Metric Invariance | 2 | 0.12 | 1.00 | .004 | .000 (.000-.000) | .000 | .000 |
|  | Scalar Invariance | 4 | 9.24 | .979 | .028 | .051 (.002-.093) | .051 | -.021 |
| Age 13 Solicitation and Rule-setting | Configural Invariance | 86 | 193.09 | .948 | .045 | .050 (.041-.058) |  |  |
|  | Metric Invariance | 95 | 206.07 | .947 | .049 | .048 (.040-.056) | -.002 | .001 |
|  | Scalar Invariance | 104 | 235.28 | .937 | .052 | .050 (.043-.058) | .002 | .01 |
| Age 13 Perceptions of Warmth, Neglect and Overcontrol  (Perceived from fathers) | Configural Invariance | 232 | 541.64 | .905 | .068 | .054 (.049-.060) |  |  |
|  | Metric Invariance | 246 | 573.98 | .899 | .071 | .054 (.049-.059) | .000 | -.006 |
|  | Scalar Invariance | 260 | 634.64 | .884 | .072 | .056 (.051-.061) | .002 | -.015 |
| Age 13 Perceptions of Warmth, Neglect and Overcontrol  (Perceived from mothers) | Configural Invariance | 234 | 458.55 | .914 | .059 | .044 (.039-.049) |  |  |
|  | Metric Invariance | 248 | 526.50 | .893 | .070 | .047 (.042 -.052) | .003 | -.021 |
|  | Scalar Invariance | 262 | 633.02 | .857 | .074 | .053 (.049-.058) | .006 | -.036 |
|  | Configural Invariance | 8 | 14.92 | .989 | .023 | .042 (.000-.072) |  |  |
| Age 13 Disclosure^a^ | Metric Invariance | 12 | 17.06 | .992 | .027 | .029 (.000-.056) | -.013 | .003 |
|  | Scalar Invariance | 16 | 78.60 | .899 | .071 | .088 (.070-.108) | .059 | -.093 |
|  | Configural Invariance | 0 | 0.00 | 1.00 | .000 | .000 (.000-.000) |  |  |
| Age 15 Psychological Control | Metric Invariance | 2 | 0.85 | 1.00 | .010 | .000 (.000-.068) | .000 | .000 |
|  | Scalar Invariance | 4 | 2.27 | 1.00 | .015 | .000 (.002-.052) | .000 | .000 |
| Age 15 Solicitation and Rule-setting | Configural Invariance | 86 | 280.11 | .922 | .059 | .069 (.061-.077) |  |  |
|  | Metric Invariance | 95 | 284.61 | .924 | .060 | .065 (.057-.073) | -.004 | .002 |
|  | Scalar Invariance | 104 | 327.53 | .911 | .064 | .067 (.060-.075) | 002 | -.013 |
| Age 15 Perceptions of Warmth, Neglect and Control  (Perceived from fathers) | Configural Invariance | 232 | 643.73 | .915 | .071 | .064 (.058-.069) |  |  |
|  | Metric Invariance | 246 | 682.91 | .910 | .073 | .064 (.059-.069) | .000 | -.005 |
|  | Scalar Invariance | 260 | 765.71 | .896 | .076 | .067 (.062-.072) | .003 | -.014 |
| Age 15 Perceptions of Warmth, Neglect and Control  (Perceived from mothers)^b^ | Configural Invariance | 230 | 523.10 | .914 | .063 | .052 (.047-.057) |  |  |
|  | Metric Invariance | 244 | 598.35 | .896 | .071 | .055 (.050 -.060) | .003 | -.018 |
|  | Scalar Invariance | 258 | 758.35 | .854 | .078 | .064 (.059-.069) | .009 | -.042 |
|  | Configural Invariance | 8 | 19.75 | .983 | .026 | .056 (.027-.085) |  |  |
| Age 15 Disclosure^a^ | Metric Invariance | 12 | 35.65 | .966 | .051 | .064 (.042-.088) | .008 | -.017 |
|  | Scalar Invariance | 16 | 83.22 | .903 | .078 | .094 (.076-.113) | .030 | -.063 |
|  | Configural Invariance | 0 | 0.00 | 1.00 | .000 | .000 (.000-.000) |  |  |
| Age 16 Psychological Control | Metric Invariance | 2 | 1.12 | 1.00 | .012 | .000 (.000-.074) | .000 | .000 |
|  | Scalar Invariance | 4 | 1.52 | 1.00 | .013 | .051 (.000-.040) | .051 | .000 |
| Age 16 Solicitation and Rule-setting | Configural Invariance | 86 | 343.35 | .919 | .055 | .080 (.072-.089) |  |  |
|  | Metric Invariance | 95 | 379.35 | .911 | .066 | .087 (.078-.096) | .007 | -.008 |
|  | Scalar Invariance | 104 | 414.85 | .903 | .069 | .080 (.073-.088) | -.007 | -.008 |
|  | Configural Invariance | 8 | 15.83 | .989 | .024 | .046 (.012-.076) |  |  |
| Age 16 Disclosure^a^ | Metric Invariance | 12 | 29.91 | .975 | .041 | .057 (.033-.081) | .011 | -.014 |
|  | Scalar Invariance | 16 | 78.67 | .914 | .070 | .092 (.073-.112) | .035 | -.061 |

*Not*e. ^a^ Reversed items of the disclosure scale (i.e., third and fifth items) were allowed to covary. ^b^ Second and fifth items and first and third items of the perception of neglect subscale were allowed to covary and the covariance between the perception of warmth and control was fixed to 0.

**Table S3**

*Item–Total Correlations for Psychological Control Items at Ages 13, 15, and 16*

| Age | Item | Full Sample | More Individualistic Countries | More Collectivistic Countries |
| --- | --- | --- | --- | --- |
| 13 | Item 1 | .43 | .46 | .38 |
|  | Item 2 | .43 | .44 | .39 |
|  | Item 3 | .46 | .50 | .41 |
| 15 | Item 1 | .45 | .40 | .45 |
|  | Item 2 | .43 | .36 | .47 |
|  | Item 3 | .48 | .42 | .50 |
| 16 | Item 1 | .46 | .42 | .48 |
|  | Item 2 | .44 | .42 | .43 |
|  | Item 3 | .47 | .48 | .45 |

*Note*. Item–total correlations are based on the correlation between each item and the total score excluding that item.

**Table S4**

*Inter-item Correlations of Control Subscale of Parental Acceptance-Rejection/Control Questionnaire-Short Form*

| **Items** |  | **1** | | **2** | | **3** | | **4** | | **5** | |  |
| --- | --- | --- | --- | --- | --- | --- | --- | --- | --- | --- | --- | --- |
| 1. Item 1^a^ |  | − |  | .489 | *** | .319 | *** | -.065 |  | .234 | *** |  |
| 2. Item 2 |  | .317 | *** | − |  | **.403** | ******* | .010 |  | **.340** | ******* |  |
| 3. Item 3 |  | .192 | *** | **.315** | ******* | − |  | .090 | ** | **.387** | ******* |  |
| 4. Item 4 (reversed)^a^ |  | .087 | ** | .020 |  | .058 |  | − |  | .098 | ** |  |
| 5. Item 5 |  | .104 | ** | **.286** | ******* | **.315** | ******* | .031 |  | − | |  |

*Note. ^a^* excluded items. The values above the diagonal are for adolescent report for fathers; the values below the diagonal are for adolescent report for mothers. Correlations among included items are displayed in bold.

** *p* < .01, *** *p* < .001.

**Table S5**

|  | Adolescent report for fathers | | |  | | Adolescent report for mothers | | |
| --- | --- | --- | --- | --- | --- | --- | --- | --- |
|  | F1 |  | F2 |  | F1 | |  | F2 |
| Item 1^a^ | .701 |  |  |  | .570 | |  |  |
| Item 2 | .788 |  |  |  | .742 | |  |  |
| Item 3 | .734 |  |  |  | .700 | |  |  |
| Item 4 (reversed)^a^ |  |  | .912 |  |  | |  | .915 |
| Item 5 | .661 |  |  |  | .631 | |  |  |
| Eigenvalue | 2.06 |  | 1.066 |  | 1.788 | |  | 1.010 |
| Explained Variance (%) | 41.92 |  | 21.32 |  | 35.76 | |  | 20.19 |

*Note*. *^a^* excluded items. F1 = Factor 1, F2 = Factor 2. No rotation method was used.

When varimax rotation was used, item 1 was cross-loaded for adolescent report for mothers

(i.e., .484 for F1 and .417 for F2).

**Table S6**

*Item–Total Correlations for Perception of Overcontrol Items at Ages 13 and 15*

| Age | Item | Adolescent Report for Fathers | | | Adolescent Report for Mothers | | |
| --- | --- | --- | --- | --- | --- | --- | --- |
|  |  | Full Sample | More Individualistic Countries | More Collectivistic Countries | Full Sample | More Individualistic Countries | More Collectivistic Countries |
| 13 | Item 1 | .32 | .50 | .20 | .24 | .40 | .12 |
|  | Item 2 | .38 | .38 | .41 | .31 | .28 | .37 |
|  | Item 3 | .36 | .35 | .37 | .29 | .28 | .31 |
| 15 | Item 1 | .45 | .58 | .32 | .37 | .53 | .23 |
|  | Item 2 | .48 | .51 | .45 | .40 | .40 | .41 |
|  | Item 3 | .43 | .46 | .40 | .37 | .36 | .43 |

*Note*. Item–total correlations are based on the correlation between each item and the total score excluding that item.

**Table S7**

*Bivariate Correlations of Study Variables with Adolescent Gender and Parental Education by Cultural Orientation*

|  | **More Individualistic Countries** | | | | **More Collectivistic Countries** | | | |
| --- | --- | --- | --- | --- | --- | --- | --- | --- |
| **Variable** | Adolescent  Gender | | Parental Education | | Adolescent Gender | | Parental Education | |
| 1. Age 13 Psychological Control | -.131 | ** | -.138 | ** | -.114 | * | -.077 |  |
| 2. Age 13 Solicitation | .130 | ** | .033 |  | .079 |  | .048 |  |
| 3. Age 13 Rule-setting | .143 | ** | -.025 |  | .189 | *** | .025 |  |
| 4. Age 13 Perception of Warmth | -.063 |  | .070 |  | .002 |  | -.016 |  |
| 5. Age 13 Perception of Neglect | -.028 |  | .025 |  | -.033 |  | .013 |  |
| 6. Age 13 Perception of Overcontrol | -.062 |  | -.236 | *** | -.039 |  | -.061 |  |
| 7. Age 13 Disclosure | .078 |  | -.023 |  | .119 | ** | .021 |  |
| 8. Age 15 Psychological Control | -.053 |  | -.033 |  | -.054 |  | -.046 |  |
| 9. Age 15 Solicitation | .125 | ** | -.028 |  | .002 |  | -.006 |  |
| 10. Age 15 Rule-setting | .179 | *** | -.056 |  | .064 |  | .113 | * |
| 11. Age 15 Perception of Warmth | -.079 |  | .004 |  | -.050 |  | .066 |  |
| 12. Age 15 Perception of Neglect | .055 |  | .063 |  | -.047 |  | -.060 |  |
| 13. Age 15 Perception of Overcontrol | -.085 |  | -.207 | *** | .004 |  | -.039 |  |
| 14. Age 15 Disclosure | .131 | ** | -.098 | * | .053 |  | .044 |  |
| 15. Age 16 Psychological Control | -.101 | * | -.079 |  | -.041 |  | -.040 |  |
| 16. Age 16 Solicitation | .121 | ** | .044 |  | .027 |  | -.014 |  |
| 17. Age 16 Rule-setting | .140 | ** | -.157 | ** | .129 | ** | .098 | * |
| 18. Age 16 Disclosure | .172 | *** | -.050 |  | .234 | *** | -.027 |  |

*Note*. Adolescent gender was dummy coded (0 = male, 1 = female).

* *p* < .05, ** *p* < .01, *** *p* < .001.

**Table S8**

*The Results of the Path Model for the Overall Sample (Including Nonsignificant Paths)*

|  | Overall | |
| --- | --- | --- |
|  | *β* | *SE* |
| Stability Paths |  |  |
| Age 15 Solicitation → Age 13 Solicitation | **.368** | **.031** |
| Age 16 Solicitation → Age 13 Solicitation | **.210** | **.029** |
| Age 16 Solicitation → Age 15 Solicitation | **.465** | **.030** |
| Age 15 Rule-setting → Age 13 Rule-setting | **.392** | **.031** |
| Age 16 Rule-setting → Age 13 Rule-setting | **.107** | **.032** |
| Age 16 Rule-setting → Age 15 Rule-setting | **.520** | **.031** |
| Age 15 Psychological Control → Age 13 Psychological Control | **.353** | **.032** |
| Age 16 Psychological Control → Age 13 Psychological Control | **.169** | **.033** |
| Age 16 Psychological Control → Age 15 Psychological Control | **.415** | **.033** |
| Age 15 Perception of Warmth → Age 13 Perception of Warmth | **.328** | **.033** |
| Age 15 Perception of Neglect → Age 13 Perception of Neglect | **.376** | **.030** |
| Age 15 Perception of Overcontrol → Age 13 Perception of Overcontrol | **.439** | **.029** |
| Age 15 Disclosure → Age 13 Disclosure | **.237** | **.040** |
| Age 16 Disclosure → Age 13 Disclosure | **.136** | **.028** |
| Age 16 Disclosure → Age 15 Disclosure | **.424** | **.038** |
| Cross-lagged paths |  |  |
| Age 13 Solicitation → Age 15 Perception of Warmth | **.113** | **.033** |
| Age 13 Rule-setting → Age 15 Perception of Warmth | -.003 | .032 |
| Age 13 Psychological Control → Age 15 Perception of Warmth | -.047 | .028 |
| Age 13 Solicitation → Age 15 Perception of Neglect | -.059 | .032 |
| Age 13 Rule-setting → Age 15 Perception of Neglect | .032 | .031 |
| Age 13 Psychological Control → Age 15 Perception of Neglect | **.117** | **.028** |
| Age 13 Solicitation → Age 15 Perception of Overcontrol | .017 | .030 |
| Age 13 Rule-setting → Age 15 Perception of Overcontrol | .035 | .030 |
| Age 13 Psychological Control → Age 15 Perception of Overcontrol | **.104** | **.031** |
| Age 13 Solicitation → Age 15 Disclosure | .070 | .037 |
| Age 13 Rule-setting → Age 15 Disclosure | .000 | .032 |
| Age 13 Psychological Control → Age 15 Disclosure | -.048 | .032 |
| Age 13 Perception of Warmth → Age 15 Disclosure | .062 | .040 |
| Age 13 Perception of Neglect → Age 15 Disclosure | .002 | .037 |
| Age 13 Perception of Overcontrol→ Age 15 Disclosure | .004 | .028 |
| Age 15 Solicitation → Age 16 Disclosure | .061 | .033 |
| Age 15 Rule-setting → Age 16 Disclosure | -.012 | .030 |
| Age 15 Psychological Control → Age 16 Disclosure | .018 | .030 |
| Age 15 Perception of Warmth → Age 16 Disclosure | .036 | .035 |
| Age 15 Perception of Neglect → Age 16 Disclosure | .003 | .033 |
| Age 15 Perception of Overcontrol→ Age 16 Disclosure | **-.104** | **.029** |

*Note.* Statistically significant paths (*p* < .05) are displayed in bold.

**Table S9**

*Standardized Path Coefficients between Covariates and Study Variables for the Overall Sample*

|  | Overall | |
| --- | --- | --- |
|  | *β* | *SE* |
| Adolescent Gender **→** |  |  |
| Age 13 Psychological Control | **-.108** | **.062** |
| Age 13 Solicitation | **.107** | **.063** |
| Age 13 Rule-setting | **.161** | **.062** |
| Age 13 Perception of Warmth | -.037 | .063 |
| Age 13 Perception of Neglect | -.023 | .063 |
| Age 13 Perception of Overcontrol | -.055 | .062 |
| Age 13 Disclosure | **.094** | **.063** |
| Age 15 Psychological Control | -.009 | .061 |
| Age 15 Solicitation | .033 | .060 |
| Age 15 Rule-setting | **.069** | **.060** |
| Age 15 Perception of Warmth | **-.073** | **.059** |
| Age 15 Perception of Neglect | .037 | .057 |
| Age 15 Perception of Overcontrol | -.019 | .058 |
| Age 15 Disclosure | **.067** | **.061** |
| Age 16 Psychological Control | -.024 | .058 |
| Age 16 Solicitation | .021 | .042 |
| Age 16 Rule-setting | **.062** | **.054** |
| Age 16 Disclosure | **.143** | **.054** |
| Parental Education **→** |  |  |
| Age 13 Psychological Control | **-.134** | **.007** |
| Age 13 Solicitation | .024 | .008 |
| Age 13 Rule-setting | -.008 | .008 |
| Age 13 Perception of Warmth | .051 | .008 |
| Age 13 Perception of Neglect | .013 | .007 |
| Age 13 Perception of Overcontrol | **-.161** | **.008** |
| Age 13 Disclosure | .003 | .007 |
| Age 15 Psychological Control | -.025 | .008 |
| Age 15 Solicitation | -.038 | .008 |
| Age 15 Rule-setting | .008 | .007 |
| Age 15 Perception of Warmth | .034 | .008 |
| Age 15 Perception of Neglect | .000 | .008 |
| Age 15 Perception of Overcontrol | -.049 | .007 |
| Age 15 Disclosure | -.033 | .007 |
| Age 16 Psychological Control | -.029 | .007 |
| Age 16 Solicitation | .021 | .006 |
| Age 16 Rule-setting | **-.086** | **.007** |
| Age 16 Disclosure | -.047 | .007 |

*Note.* Statistically significant paths (*p* < .05) are displayed in bold.

**Table S10**

*Covariances for the Overall Sample*

|  | Overall | |
| --- | --- | --- |
|  | *β* | *SE* |
| Age 13 |  |  |
| Solicitation ↔ Rule-setting | **.425** | **.035** |
| Solicitation ↔ Psychological Control | -.033 | .032 |
| Rule-setting ↔ Psychological Control | .000 | .031 |
| Solicitation ↔ Perception of Warmth | **.303** | **.034** |
| Solicitation ↔ Perception of Neglect | **-.179** | **.032** |
| Solicitation ↔ Perception of Overcontrol | **.069** | **.030** |
| Rule-setting ↔ Perception of Warmth | **.228** | **.037** |
| Rule-setting ↔ Perception of Neglect | **-.146** | **.035** |
| Rule-setting ↔ Perception of Overcontrol | **.163** | **.032** |
| Psychological Control ↔ Perception of Warmth | **-.186** | **.033** |
| Psychological Control ↔ Perception of Neglect | **.303** | **.035** |
| Psychological Control ↔ Perception of Overcontrol | **.247** | **.032** |
| Perception of Warmth ↔ Perception of Neglect | **-.554** | **.041** |
| Perception of Warmth ↔ Perception of Overcontrol | **.121** | **.031** |
| Perception of Neglect ↔ Perception of Overcontrol | **.102** | **.031** |
| Solicitation ↔ Disclosure | **.434** | **.035** |
| Rule-setting ↔ Disclosure | **.342** | **.033** |
| Psychological Control ↔ Disclosure | **-.168** | **.032** |
| Perception of Warmth ↔ Disclosure | **.471** | **.041** |
| Perception of Neglect ↔ Disclosure | **-.310** | **.046** |
| Perception of Overcontrol ↔ Disclosure | .059 | .031 |
| Age 15 |  |  |
| Solicitation ↔ Rule-setting | **.356** | **.031** |
| Solicitation ↔ Psychological Control | -.059 | .032 |
| Rule-setting ↔ Psychological Control | **.108** | **.028** |
| Solicitation ↔ Perception of Warmth | **.383** | **.034** |
| Solicitation ↔ Perception of Neglect | **-.347** | **.033** |
| Solicitation ↔ Perception of Overcontrol | .003 | .028 |
| Rule-setting ↔ Perception of Warmth | **.137** | **.031** |
| Rule-setting ↔ Perception of Neglect | **-.118** | **.027** |
| Rule-setting ↔ Perception of Overcontrol | **.125** | **.026** |
| Psychological Control ↔ Perception of Warmth | **-.259** | **.033** |
| Psychological Control ↔ Perception of Neglect | **.250** | **.031** |
| Psychological Control ↔ Perception of Overcontrol | **.280** | **.030** |
| Perception of Warmth ↔ Perception of Neglect | **-.588** | **.038** |
| Perception of Warmth ↔ Perception of Overcontrol | .067 | .031 |
| Perception of Neglect ↔ Perception of Overcontrol | **.144** | **.029** |
| Solicitation ↔ Disclosure | **.414** | **.033** |
| Rule-setting ↔ Disclosure | **.273** | **.030** |
| Psychological Control ↔ Disclosure | **-.167** | **.032** |
| Perception of Warmth ↔ Disclosure | **.402** | **.035** |
| Perception of Neglect ↔ Disclosure | **-.333** | **.033** |
| Perception of Overcontrol ↔ Disclosure | **-.079** | **.028** |
| Age 16 |  |  |
| Solicitation ↔ Rule-setting | **.352** | **.026** |
| Solicitation ↔ Psychological Control | **-.088** | **.04** |
| Rule-setting ↔ Psychological Control | **.091** | **.026** |
| Solicitation ↔ Disclosure | **.347** | **.026** |
| Rule-setting ↔ Disclosure | **.272** | **.025** |
| Psychological Control ↔ Disclosure | **-.139** | **.025** |

*Note.* Statistically significant paths (*p* < .05) are displayed in bold.
